# Treatment of landfill leachate using ASBR combined with zeolite adsorption technology

**DOI:** 10.1007/s13205-016-0513-8

**Published:** 2016-09-07

**Authors:** Chi Kim Lim, Ta Wee Seow, Chin Hong Neoh, Muhamad Hanif Md Nor, Zaharah Ibrahim, Ismail Ware, Siti Hajar Mat Sarip

**Affiliations:** 1 Department of Construction Management, Faculty of Technology Management and Business, Universiti Tun Hussein Onn Malaysia, 86400 Parit Raja Batu Pahat, Johor Malaysia; 2 Centre for Environmental Sustainability and Water Security, Universiti Teknologi Malaysia, 81310 Skudai, Johor Malaysia; 3 Department of Biosciences and Health Sciences, Faculty of Biosciences and Medical Engineering, Universiti Teknologi Malaysia, 81310 Skudai, Johor Malaysia; 4 Institute of Bioproduct Development, Universiti Teknologi Malaysia, 81310 Skudai, Johor Malaysia

**Keywords:** Landfill leachate, Sequencing batch reactor, Ammoniacal nitrogen, COD, Zeolite, Adsorption

## Abstract

Sanitary landfilling is the most common way to dispose solid urban waste; however, improper landfill management may pose serious environmental threats through discharge of high strength polluted wastewater also known as leachate. The treatment of landfill leachate to fully reduce the negative impact on the environment, is nowadays a challenge. In this study, an aerobic sequencing batch reactor (ASBR) was proposed for the treatment of locally obtained real landfill leachate with initial ammoniacal nitrogen and chemical oxygen demand (COD) concentration of 1800 and 3200 mg/L, respectively. ASBR could remove 65 % of ammoniacal nitrogen and 30 % of COD during seven days of treatment time. Thereafter, an effective adsorbent, i.e., zeolite was used as a secondary treatment step for polishing the ammoniacal nitrogen and COD content that is present in leachate. The results obtained are promising where the adsorption of leachate by zeolite further enhanced the removal of ammoniacal nitrogen and COD up to 96 and 43 %, respectively. Furthermore, this combined biological–physical treatment system was able to remove heavy metals, i.e. aluminium, vanadium, chromium, magnesium, cuprum and plumbum significantly. These results demonstrate that combined ASBR and zeolite adsorption is a feasible technique for the treatment of landfill leachate, even considering this effluent’s high resistance to treatment.

## Introduction

Population and industrial growth, technological advancements, higher living standards, changes in the productivity and consumption habits has been leading to the rapid increases in both the municipal and industrial solid waste production (Schiopu and Gavrilescu 2010). The sanitary landfill method for the ultimate disposal of solid waste material continues to be widely accepted and used as this is a relatively simple procedure with low cost (Eggen et al. 2010). Comparative studies of the various possible methods to eliminate solid urban waste such as incineration, composting, landfilling and so on have shown that the cheapest in terms of exploitation and capital costs, is landfilling (Renou et al. 2008). By nature, sanitary landfill is defined as a physically, chemically and biologically complex heterogenous system where it is resistant towards composition and compaction, temperature, moisture content as well as seasonal variations (Kylefors et al. 2003). However, landfills require proper environmental monitoring during their set-up, operation and long-term post-closure period due to the generation of leachate (a very complex wastewater) which can potentially contaminate nearby surface and ground water if left untreated (Ahmed and Lan 2012). Even if a landfill site is closed, contaminated leachate will continue to produce at the landfill site and this process could last for 30–50 years (Ngo et al. 2008). The landfill leachate would contaminate the ground water and surface water supply, and harmful to human health when migrating from the landfill and enters the surrounding lands and water. Based on the survey by the United States Environmental Protection Agency (USEPA), there are around 55,000 landfills in the USA, approximately 75 % of which are polluting groundwater. The case of water pollution by landfill leachate has also been reported globally, especially in European countries, Australia and China (Ngo et al. 2008). Landfill leachate is generated as a result of the rainwater percolation through the wastes, biochemical, chemical and physical reactions and inherent moisture content of the waste themselves (Renou et al. 2008). Leachate may contain a large amount of organic matter which is biodegradable but also refractory to biodegradation, where the main group consists of humic-type constituents, as well as ammonia–nitrogen, heavy metals (e.g. copper, iron, zinc, lead, manganese, etc.), chlorinated organic and inorganic salts (e.g. chloride, sulfate, sodium, etc.), toxic materials such as xenobiotic organic compounds, depends on waste type and compaction, landfill hydrology, climate as well as landfill age (Baig et al. 1999; Renou et al. 2008). There are three types of leachate which have been classified according to the landfill age as tabulated in Table 1. As the landfill age increased, this will result in the decrease of organic concentration and increase of ammonia nitrogen concentration in landfill leachate (Kulikowska and Klimiuk 2008). Landfill leachate from old sites usually contain high amount of ammonia as a result from the hydrolysis and fermentation of nitrogen containing fractions of biodegradable refuse substrates (Cheung et al. 1997). The correlation between the age of the landfill and the organic compounds composition may provide useful information to choose a suited treatment process.

**Table 1:** Landfill leachate classification by age (Alvarez-Vazquez et al. 2004)

	Young	Medium	Old
Age (year)	<1	1–5	>5.0
pH	<6.5	6.5–7.5	>7.5
COD (g/L)	>15	3.0–1.5	<3.0
BOD_5_/COD	0.5–1	0.1–0.5	<0.1
TOC/COD	<0.3	0.3–0.5	>0.5
Ammonium nitrogen (mg/L)	<400	400	>400
Heavy metals (mg/L)	>2.0	<2.0	<2.0
Organic compound	80 % VFA	5–30 % VFA + HA + FA	HA + FA

*COD* chemical oxygen demand, *BOD* biological oxygen demands—5 days, *TOC* total organic carbon, *VFA* volatile fat acids, *HA* humic acid, *FA* fulvic acid

The removal of organic material in terms of COD and ammonium from the leachate is always the usual prerequisite before leaving the leachate enters the natural water bodies. Numerous studies have been conducted for the treatment of landfill leachate using different approaches such as photoelectrooxidation, modified sequencing batch reactor, microalgae, vermiconversion and so on (Bakar et al. 2015; Miao et al. 2015; Muller et al. 2015; Richards et al. 2013; Wang et al. 2013). Thus, in this study, the application of biological approach for the treatment of landfill leachate will be investigated since it is more effective, environmental friendly and cost-effective, a locally obtained bacterial strain capable of treating landfill leachate will be applied in a designated ASBR system for the treatment of landfill leachate in terms of ammoniacal nitrogen, COD and heavy metal removal.

## Materials and methods

### Microorganism

The microorganism used in this study was a single bacteria strain, i.e. *Brevibacillus panacihumi* strain ZB1 which was obtained from a local textile wastewater treatment plant.

### Landfill leachate source

The leachate sample was obtained from a locally landfill site located in Johor, Malaysia. The sample was then sterilised by autoclave at 121 °C at the pressure of 101.3 kPa for 15 minutes.

### Isolation and screening of leachate degrader

A total of five bacterial strains were isolated from landfill leachate itself using streak plate method. The culture was then used as inoculum (10 % v/v) for the treatment of leachate sample in terms of ammoniacal nitrogen removal under shaking condition (150 rpm) at 37 °C for 24 hours. Besides using isolated bacterial strains, the treatment performance of leachate sample was also analysed by using known bacterial strains, such as *Brevibacillus panacihumi* strain ZB1*, Lysinibacillus fusiformis* strain ZB2 both obtained from the local textile treatment plant (Bay et al. 2013), and *Enterococcus faecalis* strain ZL isolated from local palm oil mill effluent (Lim et al. 2013).

### Analytical methods

The ammoniacal nitrogen (Nessler method) and COD (reactor digestion method) were determined by HACH DR 6000 spectrophotometer. Inductively coupled plasma-mass spectrometry (ICP-MS, Perkin Elmer Elan 6100) was applied for determination of the heavy metals in this work. The ICP-MS was operated using argon gas as carrier gas with gas flow of 0.435 L/min. The landfill leachate wastewater before and after treatment were filtered using 0.2 µm membrane and acidified to pH 2 with HNO_3_ for metal analysis.

### Reactor set-up

**Fig. 1:**
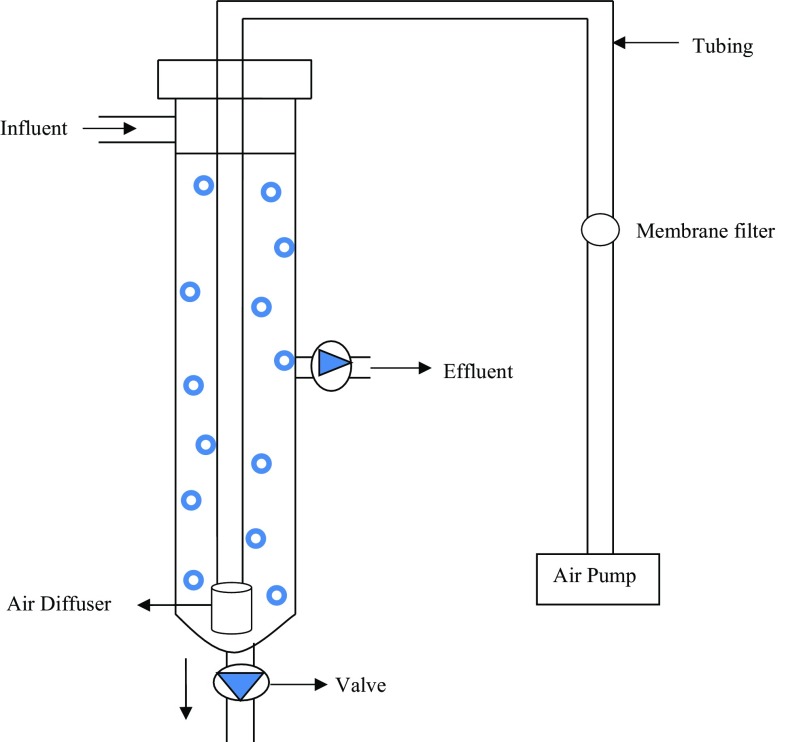
Schematic diagram of the designed ASBR system

A fabricated lab-scale glass reactor with internal diameter of 3 cm and height of 72 cm; with working volume of 300 mL was used in this study (Fig. 1). Air was introduced using a fine air bubble diffuser and an air pump (RS-248A aquarium air pump) located at the bottom of the reactor with superficial air upflow velocity of 1.0–1.2 cm/s to provide aeration to the system throughout the entire treatment process. The inoculum (10 % v/v) was acclimatised with the leachate sample for 10 days prior to the actual treatment. During acclimatisation period, a total of 150 mL effluent was being washed out every 24 hours leaving half of the content in the reactor, giving 50 % volumetric exchange rate. The reactor was then filled in with another 150 mL of fresh leachate sample with 10 % v/v of inoculum to ensure the sustainability of the biomass formed. After ten days of acclimatisation period, the treatment was carried out by analysing the ammoniacal nitrogen, COD and heavy metal removal by this particular reactor system at regular intervals for 7 days. All the experiments were carried out in triplicates and average values were used for further calculations.

### Batch adsorption experiment

After seven days of biological degradation by the reactor system, adsorption experiment (physical removal) was carried out using 10 % of zeolite to adsorb the effluent discharged from the system. Zeolite (mineral form: clinoptilolite; empirical formula: (Ca,K_2_,Na_2_,Mg)_4_Al_3_Si_40_O_98_24H_2_O; Si/Al 4.8–5.4; pH 6.6–7.2; particle size 2.5–5.0 mm) used in this study was originated from Czech Republic. Experiment was performed in an incubator shaker (Hotech, 702) at 150 rpm and room temperature for 24 hours. The sample was then centrifuged at 4000 rpm for 15 minutes at 4 °C followed by the ammoniacal nitrogen, COD and heavy metal removal analysis.

## Results and discussion

### Characterisation of landfill leachate

The characterisation of the landfill leachate sample was carried out in terms of its pH, COD and ammoniacal nitrogen (Table 2).

**Table 2:** Characterisation of landfill leachate

Parameter	Value
pH	9.66
COD	3200 ± 100 mg/L
Ammoniacal nitrogen	1800 ± 50 mg/L

### Isolation and screening of leachate degrader

Isolation and screening of bacteria are important steps in studying and evaluating the biodegradation potential of the microorganisms in various organic pollutants. In this study, a total of five pure cultures of bacteria was successfully isolated from the leachate sample for the treatment of landfill leachate. Furthermore, three other known bacterial cultures (*Brevibacillus panacihumi* strain ZB1, *Lysinibacillus fusiformis* strain ZB2 and *Enterococcus faecalis* strain ZL) were also included in the selection process. In general, the ammoniacal nitrogen removal capacity obtained ranged from 3 to 23 % after being incubated under shaking condition at 37 °C for 24 hours. The highest removal was 23 % (*B. panacihumi* strain ZB1). The lower ammoniacal nitrogen removal efficiency (3–14 %) was observed for the other isolated bacteria, this may be due to that they do not possess the abilities like *B. panacihumi* strain ZB1 has, where it is an aerobic bacteria that works well under aerobic condition. In addition, the strain belongs to *Brevibacillus* genus which is able to reduce nitrate (Li et al. 2015). Therefore, this bacterial strain was chosen for further studies. A control experiment was also conducted using leachate sample without the addition of inoculum. The results obtained were summarised in Table 3.

**Table 3:** Ammoniacal nitrogen removal by various bacterial strains

Bacterial strain	Ammoniacal nitrogen removal after 24 h (%)
Strain A	11.7 ± 0.33
Strain B	8.5 ± 0.24
Strain C	14.3 ± 0.57
Strain D	3.7 ± 0.78
Strain E	12.6 ± 0.26
*Brevibacillus panacihumi* strain ZB1	22.8 ± 0.55
*Lysinibacillus fusiformis* strain ZB2	9.83 ± 0.28
*Enterococcus faecalis* strain ZL	14.2 ± 0.33
Control	None

### Ammoniacal nitrogen and COD removal

**Fig. 2:**
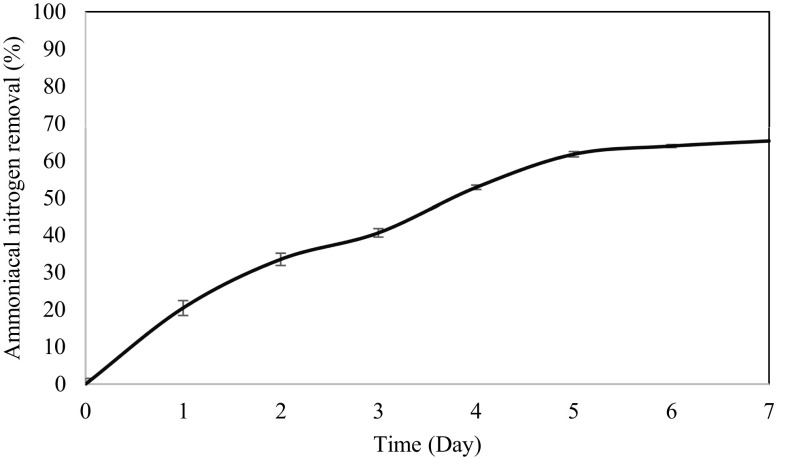
Ammoniacal nitrogen removal efficiency during 7 days of ASBR treatment system

Figure 2 shows the removal of ammoniacal nitrogen by the designed reactor system using *B. panacihumi* strain ZB1. The results obtained showed that the bacterial strain ZB1 was able to remove the ammoniacal nitrogen up to 65 % during seven days of aerobic treatment process. Since *B. panacihumi* strain ZB1 is a kind of nitrifying bacteria, nitrification will take place during the treatment process. Under aerobic condition, strain ZB1 will undergo nitrification which involves two steps, i.e. the oxidation of ammonia/ammonium to nitrite followed by the oxidation of the nitrite to nitrate (Hulle et al. 2005). The chemistry behind this process is given in the following equation:Nitrification:

1 $${\text{NH}}_{ 4}^{ + } \;{ + }\; 2 {\text{O}}_{ 2} \; \to \;{\text{NO}}_{3}^{ - } {\text{ + 2H}}^{ + } {\text{ + H}}_{ 2} {\text{O}} .$$

The effluent obtained was then adsorbed by the effective adsorbent, i.e. zeolite for 24 hours, the removal efficiency was drastically increased up to 96 % (almost complete removal was achieved). The findings obtained indicated that the addition of zeolite may be an effective alternative for upgrading the performance of the wastewater treatment plant, used as a secondary treatment step for polishing the ammoniacal nitrogen content. In fact, the application of natural and modified zeolite as ion exchanger is one of the most effective technologies used to remove various contaminants due to their high ion-exchange capacity, high specific surface areas and relatively low cost (Crini 2006). The performance of different treatments was investigated in the present study to evaluate the ammoniacal nitrogen removal efficiency from landfill leachate. A study conducted by Ozturk et al. (2005) reported that the maximum removal of ammoniacal nitrogen was 62.8 % from initial concentration of 950 mg/L. Moraes and Bertazzoli (2005) found out that the maximum removal of ammoniacal nitrogen with initial concentration of 1060 mg/L obtained by employing a flow electrochemical reactor was 49 %. Compared with the efficiency of other treatment processes, the performance of this study can be considered satisfactory. In fact, the traditional nitrogen removal process is a combination of aerobic nitrification and anaerobic denitrification catalysed by autotrophs and heterotrophs, separately (Kuenen and Robertson 1994). A higher degree of treatment performance can be expected when co-metabolic activities within a microbial community complement each other during the wastewater treatment as compared to a pure culture system. However, it should be stressed that the composition of mixed cultures may change during the treatment process, which interferes with the control of technologies using mixed cultures. On the other hand, the data that are obtained with use of pure culture system are reproducible and that the interpretation of experimental observations is easier. Also, the response of the system to changes in operational parameters can be studied as well (Pearce et al. 2003). Thus, a pure bacterial culture was chosen in this study to provide a more fundamental study for the landfill leachate treatment using a designated ASBR system.

**Fig. 3:**
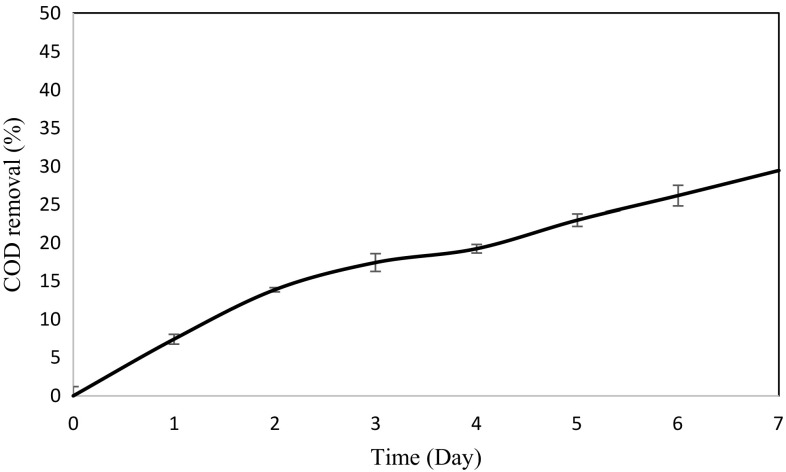
COD removal efficiency during 7 days of ASBR treatment system

As for the COD removal (Fig. 3), it is an important measure of water quality as it determines the amount of organic contamination in the wastewater. The results obtained showed that the reactor system was able to reduce the COD value from 3150 to 2224 mg/L (30 %) while further adsorbed by the zeolite (24 hours) had increased the removal efficiency up to 43 %. The organic compounds available in the wastewater are typically used as electron donors for denitrification. Even so, a considerable fraction of the COD is still oxidised aerobically due to endogenous respiration of biomass (Virdis et al. 2008). This suggested that COD was also partly removed in this study. ASBR was applied for the COD removal as the removal efficiency was higher under aerobic condition when compared to anoxic or anaerobic processes. One possible reason may be due to the bacteria oxidised the organic compounds for carbon and energy source under aerobic condition which resulted in the COD reduction. This is proven by Magnaye et al. (2009) where a preliminary study on the efficiency of nitrogen-rich simulated wastewater using two different reactors, aerobic and anaerobic was carried out. The results showed that 98 % reduction in COD was obtained in aerobic reactor, with a hydraulic retention time (HRT) of 5 hours after 11 days while 34 % reduction in COD was obtained in anaerobic reactor with the same HRT after 14 days. Further COD removal analysis using aerobic batch reactors with initial concentrations of 500, 1000, 1500, 2000 and 2500 ppm showed 71 to 87 % reduction in COD within an average time of 4 to 5 days. Another research reported by Kargi and Pamukoglu (2003) applying aerobic treatment for the pre-treated landfill leachate showed nearly 76 % COD and 23 % NH_4_-N removals after 30 hours of operation with a flow rate of 0.21 l/h and the feed COD content of 7000 mg COD/L. These findings showed that the aerobic condition is applicable for the treatment of wastewater with high COD content.

### Heavy metal analysis

The question of heavy metal content as a potential hazard is a frequently addressed concern in leachate composition. Thus, in this study, ICP-MS method is used to identify and quantify the metals present in water at trace levels. Table 4 presents the concentrations of metals in landfill leachate wastewater used in this study. The results showed that *B. panacihumi* strain ZB1 was able to reduce the concentration of aluminium, vanadium, chromium, magnesium, cuprum, and plumbum from 8 to 50 %. After seven days of aerobic treatment by the designed reactor system, the polishing step by the zeolite adsorption further enhanced the removal performance which proved that this combined biological–physical treatment is able to remove the heavy metal that presence in the wastewater significantly.

**Table 4:** Metal content in landfill leachate wastewater

Analyte	Initial concentration (parts per billion)	Removal after 7 days of treatment (%)	Removal after 7 days of ASBR treatment + 24 h of zeolite adsorption(%)
Aluminium	1381.8	50	~100
Vanadium	72.5	14	44
Chromium	1109.5	24	63
Magnesium	278.2	46	75
Cuprum	602.5	8	24
Plumbum	2413	43	85

## Conclusion

A novel system consisting of ASBR and zeolite adsorption was proposed for the treatment of landfill leachate, and achieved outstanding performance for advanced ammoniacal nitrogen removal. This system was able to remove 96 % of ammoniacal nitrogen and 43 % of COD that presence in leachate. In addition, the system also demonstrated the removal of heavy metals (aluminum, plumbum, magnesium, etc.) found in leachate. Summing up, this combined biological–physical treatment significantly removing the contaminants that exist in leachate. This process is then feasible as an option for leachate treatment.

## References

[CR1] Ahmed FN, Lan CQ (2012). Treatment of landfill leachate using membrane bioreactors: a review. Desalination.

[CR2] Alvarez-Vazquez H, Jefferson B, Judd SJ (2004). Membrane bioreactors vs conventional biological treatment of landfill leachate: a brief review. J Chem Technol Biotechnol.

[CR3] Baig S, Coulomb I, Courant P, Liechti P (1999). Treatment of landfill leachates: Lapeyrouse and Satrod case studies. Ozone Sci Eng.

[CR4] Bakar AA, Yee CM, Mahmood NZ, Abdullah N (2015). Effect on heavy metals concentration from vermiconversion of agro-waste mixed with landfill leachate. Waste Manag.

[CR5] Bay HH, Lim CK, Kee TC, Olsson G, Ware I, Chan GF, Shahir S, Ibrahim Z (2013). Biodecolourisation of Acid Orange 7 coloured by-products using an acclimatised mixed bacterial culture. Environ Sci Pollut Res.

[CR6] Cheung KC, Chu LM, Wong MH (1997). Ammonia stripping as a pretreatment for landfill leachate. Water Air Soil Pollut.

[CR7] Crini G (2006). Non-conventional low-cost adsorbents for dye removal: a review. Bioresour Technol.

[CR8] Eggen T, Moeder M, Arukwe A (2010). Municipal landfill leachates: a significant source for new and emerging pollutants. Science of Total Environment.

[CR9] Hulle SWHV, Broeck VD, Martens J, Villez D, Donckels BMR, Schelstraete G, Volcke EIP, Vanrolleghem PA (2005). Construction, start-up and operation of a continuously aerated laboratory-scale SHARON reactor in view of coupling with an Anammox reactor. Water SA.

[CR10] Kargi F, Pamukoglu MY (2003). Aerobic biological treatment of pre-treated landfill leachate by fed-batch operation. Enzym Microb Technol.

[CR11] Kuenen JG, Robertson LA (1994). Combined nitrification–denitrification processes. FEMS Microbiol Rev.

[CR12] Kulikowska D, Klimiuk E (2008). The effect of landfill age on municipal leachate composition. Bioresour Technol.

[CR13] Kylefors K, Andreas L, Lagerkvist A (2003). A comparison of small-scale, pilot-scale and large-scale tests for predicting leaching behavior of landfilled wastes. Waste Manag.

[CR14] Li G, Liang Z, An T, Zhang Z, Chen X (2015). Efficient bio-deodorization of thioanisole by a novel bacterium Brevibacillus borstelensis GIGAN1 immobilized onto different parking materials in twin biotrickling filter. Bioresour Technol.

[CR15] Lim CK, Bay HH, Aris A, Majid AZ, Ibrahim Z (2013). Biosorption and biodegradation of Acid Orange 7 by *Enterococcus faecalis* strain ZL: optimization by response surface methodological approach. Environ Sci Pollut Res.

[CR16] Magnaye FA, Gaspillo PD, Auresenia JL (2009). Biological nitrogen and cod removal of nutrient-rich wastewater using aerobic and anaerobic reactors. JWARP.

[CR17] Miao L, Wang S, Li B, Cao T, Xue T, Peng Y (2015). Advanced nitrogen removal via nitrite using stored polymers in a modified sequencing batch reactor treating landfill leachate. Bioresour Technol.

[CR18] Moraes PB, Bertazzoli R (2005). Electrodegradation of landfill leachate in a flow electrochemical reactor. Chemosphere.

[CR19] Muller GT, Giacobbo A, Chiaramonte EADS, Rodrigues MAS, Meneguzzi A, Bernardes AM (2015). The effect of sanitary landfill leachate aging on the biological treatment and assessment of photoelectrooxidation as a pre-treatment process. Waste Manag.

[CR20] Ngo HH, Guo W, Xing W (2008) Applied technologies in municipal solid waste landfill leachate treatment. In: Vigneshwaran S (ed) Water and wastewater treatment technologies, vol. 2. Eolss Publishers Co Ltd., Oxford, p 17

[CR21] Ozturk I, Altinbas M, Koyuncu I, Arikan O, Gomec-Yangin C (2003). Advanced physico-chemical treatment experiences on young municipal landfill leachates. Waste Manag.

[CR22] Pearce CI, Lloyd JR, Guthrie JT (2003). The removal of colour from textile wastewater using whole bacterial cells: a review. Dyes Pigm.

[CR23] Renou S, Givaudan JG, Poulain S, Dirassouyan F, Moulin P (2008). Landfill leachate treatment: review and opportunity. J Hazard Mater.

[CR24] Richards RG, Mullins BJ (2013). Using microalgae for combined lipid production and heavy metal removal from leachate. Ecol Model.

[CR25] Schiopu AM, Gavrilescu M (2010). Options for the treatment and management of municipal landfill leachate: common and specific issues. Clean Soil Air Water.

[CR26] Virdis B, Rabaey K, Yuan Z, Keller J (2008). Microbial fuel cells for simultaneous carbon and nitrogen removal. Water Res.

[CR27] Wang K, Wang S, Zhu R, Miao L, Peng Y (2013). Advanced nitrogen removal from landfill leachate without addition of external carbon using a novel system coupling ASBR and modified SBR. Bioresour Technol.

